# Cardiotoxic Effect Induced by F-53B via Nitric Oxide Signalling on Parkin^−/−^ Mice

**DOI:** 10.3390/toxics13110942

**Published:** 2025-10-31

**Authors:** Jun Nie, Chao Hu, Yuru Huang, Ying Ma, Liping Lu

**Affiliations:** 1 School of Engineering, Hangzhou Normal University, Hangzhou 310018, China; 2023111010076@stu.hznu.edu.cn; 2 School of Life and Environmental Sciences, Hangzhou Normal University, Hangzhou 311121, China; huchao_1003@163.com; 3 School of Public Health, Hangzhou Normal University, Hangzhou 311121, China; huangyuru2019@163.com (Y.H.); 2023111024004@stu.hznu.edu.cn (Y.M.)

**Keywords:** chlorinated polyfluoroalkyl ether sulfonic acid, parkin^−/−^ mice, cardiotoxicity, endothelial nitric oxide synthase, nitric oxide

## Abstract

A comprehensive understanding of gene-environment interactions is essential for maintaining human cardiac health, and deficiency in the key *parkin* gene exacerbates cardiac injury. Per- and polyfluoroalkyl substances (PFAS) exposure has been determined cardiotoxicity from the epidemiological perspective but the potential remained unclear. Here, we investigated the co-effects on cardiac pathological structure and function of an emerging PFAS, 6:2 chlorinated polyfluorinated ether sulfonate acid (F-53B), on male parkin^−/−^ mice at dose of 3 and 3000 μg/kg for 60 d. Mechanism was focused on the activity, phosphorylation of endothelial nitric oxide synthase (eNOS), and the content of nitric oxide (NO), vital vascular function regulating molecule. F-53B significantly increased cardiac fibrosis to 1.58- and 2.80-fold, and cardiac troponin T (cTNT) to 1.17- and 1.32-fold compared with control group, at dose of 3 and 3000 μg/kg, respectively, indicating F-53B can inhibit the normal activities of the heart and cause functional disorders. Content and phosphorylation of eNOS significantly decreased to 0.68-, 0.67-fold, and to 0.65-, 0.54-fold compared with control group, respectively. The subsequent content of NO level was also significantly decreased to 0.47- and 0.33-fold, respectively, indicating that significant co-effects of parkin deficiency and F-53B exposure on cardiac function and structural changes via eNOS/NO signalling. Our work underscores the importance of assessing cardiac risk associated with PFAS at environmentally relevant doses, especially considering environmental exposure and gene co-interaction from the perspective of F-53B and *parkin* gene.

## 1. Introduction

The ratio of 6:2 chlorinated polyfluorinated ether sulfonate acid (6:2 Cl-PFESA, F-53B) is one of the typical per- and polyfluoroalkyl substance (PFAS) substitutes for perfluorooctane sulfonic acid (PFOS), which is restricted due to adverse human health effects [1,2,3]. Research has found that PFASs and F-53B in the environment mainly result from extensive industrial use and release, extensive application of consumer goods, and improper waste disposal [4,5,6]. F-53B has been widely detected in the environment matrices [7,8], with reported concentration up to 52.0 ng/L in surface water samples [9], 33.7 ng/g dw and 3.39 ng/g dw in soil and plant samples, respectively [10]. The various exposure routes caused the increasingly reported residues in the human specimens [11] with the reported maximum concentration of the blood level being 8.64 ng/mL in the general population [2] and 5.04 μg/mL in the electroplating workers [12,13]. Human serum median concentration of F-53B was reported to be 0.7–0.9 ng/mL [14]. Numerous studies have raised health concerns about the adverse outcomes including hepatotoxicity, thyroid disruption, neurotoxicity, gut toxicity [15,16,17,18]. Recently, toxicological and epidemiological studies have showed association between PFAS exposure and cardiotoxicity [2,19,20], but limited studies have explored the cardiac effects of emerging PFAS including F-53B.

Cardiovascular disease (CVD) risk factors are complex especially with interaction of environment and gene, and understanding the effects and potential mechanism is of concern but remains to be further investigated [21]. The adverse effects identification induced by environmentally relevant dose of cardiac toxics including PFAS may be underestimated due to the lack of consideration of other factors including gene [19]. Of note, prior studies showed that environmental chemicals caused cardiac toxicity in genetically defective mice [22]. To further investigate the actual effects based on treatment mimicking the more real circumstances under PFAS exposure, research on co-effects of cardiac health is warranted, which contributes to effective risk assessment toward low-dose exposure and gene deficiency.

Parkin is reported to effectively protect against impaired cardiac function and Parkin-deficient mice are more sensitive to myocardial infarction [23]. Parkin modulates endothelial nitric oxide synthase (eNOS) signalling [24], phosphorylation eNOS leads to control vasodilation and cardiac protection via nitric oxide (NO) production [25], which is markedly abnormal in cardiac dysfunction and cardiac failure [26]. Emerging pollutants can induce vascular and cardiac myocytes injury through eNOS signal transduction [27,28]. However, the co-effects and main mechanism still need to be investigated for PFAS exposure and Parkin deficiency, especially for low dose exposure for PFAS.

In this manuscript, based on the concentration of F-53B in the human body [2], and considering that the exposure dose and time should be close to the actual exposure of the human, we decided to expose male parkin^−/−^ mice to 0, 3, and 3000 μg/kg for 60 days. Cardiac structure and fibrosis were determined for pathological injury, and the concentrations of cardiac troponin T (cTnT), brain natriuretic peptide (BNP), and myoglobin (Mb) were analyzed to evaluate cardiac function and myocardial injury caused by F53B. As for mechanism, the gene and protein expression, phosphorylation and enzyme concentration of eNOS, and NO production were detected of parkin^−/−^ mice. Our research results aim to provide evidence for the cardiotoxic effect and potential mechanism of F-53B, demonstrating that the parkin-mediated eNOS/NO pathway plays an important role in cardio dysfunction.

## 2. Materials and Methods

### 2.1. Chemicals

The 6:2 Cl-PFESA used in the experiments was obtained from the commercial product F-53B (Wellington Laboratories Inc., Guelph, ON, Canada) after purification according to previous study [29].

### 2.2. Animals and F-53B Exposure

The experiments were approved by Animal Experimentation Ethics Committee of Hangzhou Normal University (protocol number: HSD-20231115-01) [30]. Eight-month-old male parkin^−/−^ mice were selected and housed in an SPF laboratory animal environment. Ten mice were randomly assigned for each group with drinking water dissolved of F-53B (3 and 30 mg/kg for 7 d, 3 and 3000 μg/kg for 60 d). At the end of the exposure, mice were executed by spinal subluxation, and blood samples were collected from the abdominal aorta and serum was then obtained and stored at −80 °C. Heart tissue was excised and stored in 4% paraformaldehyde for HE analysis, and −80 °C for mechanism analysis. All procedures reported in the study involving animals complied with the ARRIVE guidelines.

### 2.3. Masson Staining

Heart tissues (n = 3 biological replicates) were taken in 4% paraformaldehyde for 72 h, embedded in paraffin and sectioned. Sections were stained with Masson staining kit (catalogue: HK2020, HaoKe Biotechnology Co., LTD, Hangzhou, China) and observed with a microscope (Nikon Instech Co., Ltd., Tokyo, Japan) [31]. The images were analyzed using Image J 1.54p (National Institutes of Health, Baltimore, MD, USA) software to quantify the contents of fibrous tissue with three random fields of view selected from each stained image for analysis [32,33].

### 2.4. cTnT, BNP, and Mb Contents Determination

Cardio-functions were determined using the mouse cardiac specific troponin T (cTnT) kit (ELISA) (catalogue: ml231456, mlbio, Shanghai, China), mouse brain natriuretic peptide (BNP) kit (ELISA) (catalogue: ml037594, mlbio, Shanghai, China), mouse myoglobin (Mb) kit (ELISA) (catalogue: ml063376, mlbio, Shanghai, China), following reported study [34]. To the pre-coated microtiter wells pre-coated with capture antibody, the sample, standard, and HRP-labelled detection antibody were added sequentially, incubated at 37 °C, and wash thoroughly. Absorbance was measured at 450 nm in each well using a Spark Multifunctional Microplate Tester (Tecan Group Ltd., Männedorf, Switzerland) and the concentration was calculated. Each assay was conducted with three biological and technological replicates.

### 2.5. Nitric Oxide Determination

NO levels were determined following manufacturer’s directives of nitric oxide assay kit (catalogue: S0021, Beyotime Biotech, Shanghai, China) [31]. Serum was diluted 10-fold and 50 µL of standard and serum dilutions were added to each well of a 96-well plate (catalogue: HSP9601, BIO-RAID Laboratories Inc., Hercules, CA, USA), followed by 50 µL of Griess Reagent I and II. Absorbance was measured at 540 nm in each well using a Spark Multifunctional Microplate Tester (Tecan Group Ltd., Männedorf, Switzerland). Three parallels were set up for each sample concentration.

### 2.6. eNOS and iNOS Contents Determination

The concentrations of endothelial nitric oxide synthase (eNOS) and inducible nitric oxide synthase (iNOS) were determined using commercially available ELISA kits (catalogue: ml063615 and ml057773, mlbio, Shanghai, China). Absorbance was measured at 450 nm using a Spark Multifunctional Microplate Reader (Tecan Group Ltd., Männedorf, Switzerland).

### 2.7. Quantitative Polymerase Chain Reaction (qPCR)

#### 2.7.1. The Extraction Method of Total RNA

Total RNA was extracted from the hearts of parkin^−/−^ male mice using TRIzol reagent, following the manufacturer’s protocol. 

#### 2.7.2. The Design of Primer Sequences

Primer sequences are listed in Table S1.

#### 2.7.3. qPCR Experimental Materials and Methods

cDNA synthesis was performed using Hieff^®^ III 1st Strand cDNA Synthesis SuperMix (catalogue: 11141ES60, YEASEN, Shanghai, China) by using total RNA, following the manufacturer’s protocol. Each reaction mixture contained cDNA, 0.3 μL forward and 0.3 μL reverse primers, 3.4 μL DEPC water and 5 μL Hieff^®^ qPCR SYBER GREEN Master Mix (No Rox) (catalogue: 11201ES08, YEASEN, Shanghai, China). Amplification was performed on a CFX384 Real-Time PCR System (Bio-Rad, Hercules, CA, USA) under the following conditions: at 95 °C for 5 min for one cycle; at 95 °C for 10 s, 60 °C for 20 s, 72 °C for 20 s for 40 cycles; at 65 °C for 5 s for one cycle. Relative mRNA expression levels were quantified using the 2^−ΔΔCt^ method, normalized to *GAPDH*. Experiments were conducted in three biological replicates, each with two technical replicates.

### 2.8. Western Blotting Analysis

Proteins were extracted from mice hearts using T-PER tissue protein extraction reagent (catalogue: 78510, Thermo Pierce, Waltham, MA, USA). Protein concentrations were determined using enhanced BCA protein assay kit (catalogue: P0010, Beyotime Biotechnology, Shanghai, China). Equal amounts of protein were separated by SDS-PAGE and transferred onto PVDF membranes (catalogue: IPVH00010, Millipore, Billerica, MA, USA). Membranes were blocked with 5% bovine serum albumin (BSA) (catalogue: A104912, aladdin, Shanghai, China) in TBST for 1 h at room temperature, followed by incubation with the following primary antibodies overnight at 4 °C: inducible nitric oxide synthase (iNOS, catalogue: 18985-1-AP, Proteintech, Chicago, CA, USA), eNOS (catalogue: 27120-1-AP, Proteintech, Chicago, CA, USA), Phospho-eNOS (Ser1179) Polyalonal Antibody (catalogue: 36-9100, Thermo Fisher Scientific, Waltham, MA, USA), and GAPDH (catalogue: ab9485, Abcam, Cambridge, UK). After washing, membranes were incubated with HRP-conjugated secondary antibodies for 1 h at room temperature. Protein bands were visualized using the SuperSignal™ West Dura Extended Duration Substrate (catalogue: 34075, Thermo Pierce, Waltham, MA, USA) and detected using a chemiluminescence imaging system.

### 2.9. Statistical Analysis

Statistical analysis and figure presentation were performed using GraphPad Prism 9.5 (GraphPad Software Inc., San Diego, CA, USA). Normally distributed data for the F-53B-treated and control groups were analyzed by one-way ANOVA and LSD multiple comparisons. Differences were considered statistically significant at *p* < 0.05 and marked with an asterisk.

## 3. Results and Discussion

### 3.1. F-53B Induces Cardiac Fibrosis in Parkin^−/−^ Mice

**Figure 1:**
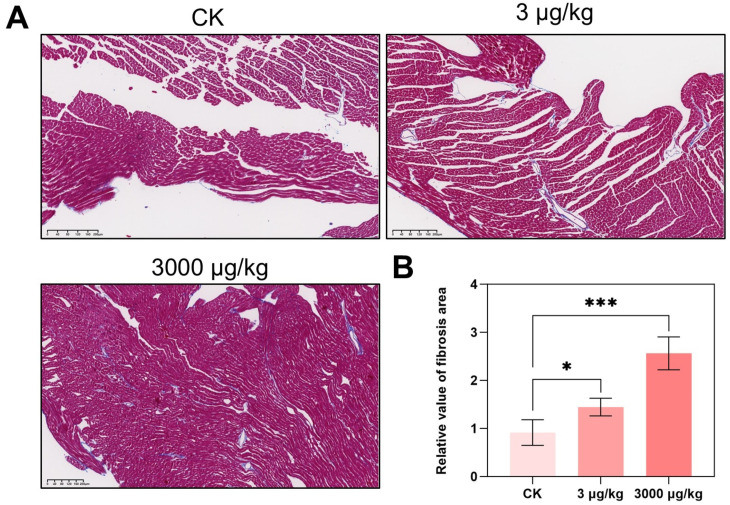
F-53B induced myocardial injury in parkin^−/−^ male mice. Parkin^−/−^ mice were exposed to 0, 3, and 3000 μg/kg F-53B for 60 d. Masson staining was used to obtain the degree of myocardial fibrosis (**A**) and to quantify the relative value of fibrosis of heart (**B**) (n = 3 biological replicates). Scale bar represented 200 μm. All data are represented as means ± SEM. * represented *p* < 0.05, and *** represented *p* < 0.001 compared with control group.

Cardiac fibrosis is the hallmark pathological feature of numerous heart diseases, including myocardial infarction, hypertrophic cardiomyopathy, dilated cardiomyopathy, diabetic cardiomyopathy, and aortic stenosis [35]. Compared with the control group, cardiac fibrosis area significantly increased by F-53B exposure (Figure 1A), reaching 1.58- and 2.80-fold at 3 and 3000 μg/kg groups, respectively (Figure 1B), which is comparable to the effects observed by groups of exposure to higher dose of 3 and 30 mg/kg for 7 days (reaching to 1.52- and 1.82-fold of control group, respectively) (Figure S1). Cardiac fibrosis is associated with impaired cardiac function [36], and serves as key indicator of structural and functional deterioration of the heart [37,38,39]. Our findings showed that F-53B induces cardiac structural damage, which likely contributes to functional impairment. Existing toxicological and epidemiological research indicates that PFAS, including PFOSA, can affect the cardiac structure and function of the heart at early life stage [40,41]; PFOA induced abnormal cardiac structure in hatchling chicken [42]. Additionally, F-53B was reported to induce potential developmental cardiac toxicity in both human embryonic stem cell model and in vivo zebrafish model, and the effects of F-53B were reported to be more robust than PFOS [37,43]. Despite of the developmental cardiotoxicity, F-53B further showed to induce cardiac fibrosis in adult male mice in this study.

### 3.2. F-53B Induces Cardiac Dysfunction in Parkin^−/−^ Mice

**Figure 2:**
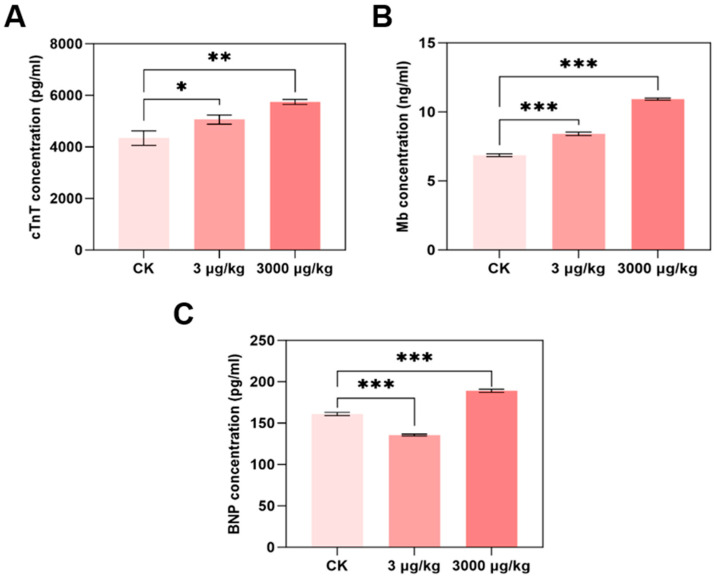
F-53B induced cardiac dysfunction in parkin^−/−^ male mice. Parkin^−/−^ male mice were exposed to 0, 3, and 3000 μg/kg F-53B for 60 d. The content of cTNT (**A**), Mb (**B**), BNP (**C**) in mice heart measured by ELISA kit (n = 3 biological replicates). All data are represented as means ± SEM. * represented *p* < 0.05, ** represented *p* < 0.01, and *** represented *p* < 0.001 compared with control group.

To further investigate cardiac function interruption by F-53B, levels of marker of cardiac function including cTnT, Mb, and BNP were determined (Figure 2A–C). Compared with the control group, cTnT levels increased significantly by 1.17- and 1.32-fold in mice exposed to 3 and 3000 μg/kg F-53B, respectively (Figure 2A). Similarly, Mb levels increased to 1.23- and 1.59-fold at the same doses (Figure 2B). BNP exhibited a biphasic response, decreasing to 0.90-fold at 3 μg/kg but increasing to 1.17-fold at 3000 μg/kg (Figure 2C).

cTnT is a clinical gold-standard biomarker of myocardial injury [44], with elevated levels reflecting cardiomyocyte necrosis and adverse cardiac events [45]. The dose-dependent increased cTNT content in parkin^−/−^ mice induced by F-53B treatment suggests progressive cardiac damage, which is consistent with previous finding [46]. Mb is a cytoplasmic oxygen transporter in skeletal and cardiac muscle [47] and regulates NO homeostasis and reactive oxygen species (ROS) detoxification [48]. The increased Mb levels with F-53B exposure indicates compensatory adaptation to increased cardiac workload and oxygen demand; heart stress increased and enhanced the ability to clear NO [49]. BNP is a diagnostic marker of heart failure [50], which is strongly associated with the severity of heart failure [51], and is released in response to ventricular stress [52] or fibrosis-induced hypertrophy [53]. These findings in the study showed BNP level decreased at 3 μg/kg and then increased at 3000 μg/kg of F-53B exposure; the biphasic BNP response may be due to that transient protective effect to reduce myocardial stress, and subsequently suppressed BNP secretion. Furthermore, higher dose of F-53B is likely to induce cardiomyocyte death and cardiac dysfunction, triggering compensatory BNP elevation, which exhibits similar effects reported for PFOS [54]. These collaborative results demonstrated that F-53B, as a PFOS alternative, disrupts cardiac function, with biomarker profiles indicating dose-dependent myocardial injury and adaptive stress responses.

### 3.3. F-53B Disrupts eNOS/iNOS/NO Signalling in Parkin^−/−^ Mice

**Figure 3:**
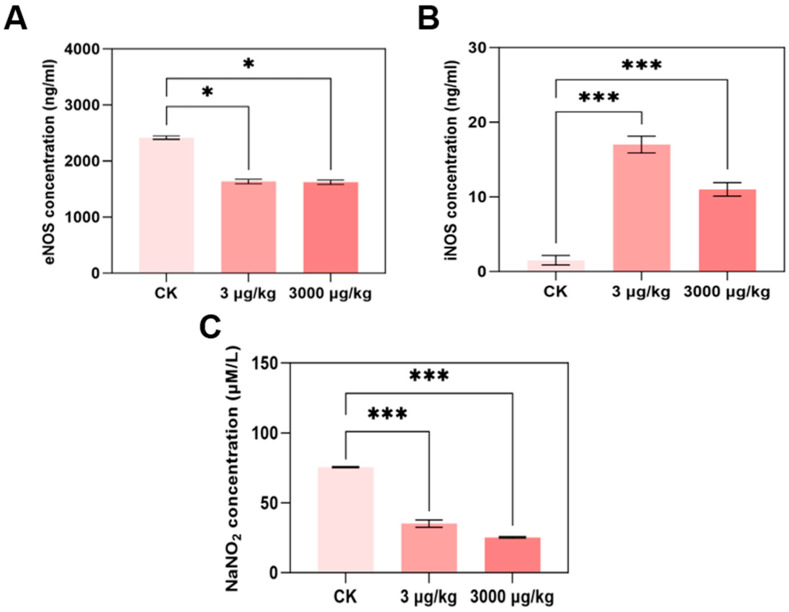
F-53B disrupted eNOS/iNOS contents in parkin^−/−^ male mice. The content of iNOS and eNOS in mice heart were measured by ELISA kit (**A**,**B**) (n = 3 biological replicates). The content of NO in mice heart was measured by nitric oxide assay kit (**C**) (n = 3 biological replicates). All data are represented as means ± SEM. * represented *p* < 0.05, *** represented *p* < 0.001 compared with control group.

After treatment with 3 and 3000 μg/kg F-53B, eNOS enzyme content was significantly decreased to 0.68- and 0.67-fold compared with the control group, respectively (Figure 3A). In contrast, the enzymatic content of iNOS was significantly increased to 11.18- and 7.24-fold (Figure 3B). The NO content of 3 and 3000 μg/kg was significantly reduced to 0.47- and 0.33-fold of control group (Figure 3C), but NO content of 3 mg/kg group was significantly reduced to 0.73-fold and increased to 1.75-fold in 30 mg/kg group (Figure S2).

NO is a primary determinant of cardiomyocyte contractility and modulates cardiac function directly [55]. Abnormal NO level is regarded as a hallmark of various human cardiac health and pan-vascular diseases, including atherosclerosis, hypertension, and myocardial infarction [56,57,58]. In terms of production mechanism, NO is mainly generated by eNOS under physiological conditions and iNOS under pathophysiological conditions in cardiomyocytes [59]. eNOS catalyzes the production of NO from L-arginine [60] and plays a critical role in cardioprotection [61,62]. eNOS disruption is a well-established pathogenic factor of CVD [63,64], and the role of the eNOS/NO pathway has also been confirmed in cardiac and vascular toxicity induced by environmental chemicals including polycyclic aromatic hydrocarbons [27]. Additionally, increased iNOS levels are related to myocardial infarction [65,66]. In this study, the result showed F-53B decreased the content of eNOS and increased iNOS content, leading to the decrease in NO level mainly derived from eNOS at 3 and 3000 μg/kg group. However, for 30 mg/kg group, it is more likely to induce pathophysiological conditions and trigger iNOS-derived NO, leading to the increased level of NO. These findings suggest that exposure to environmentally relevant concentrations of F-53B induce abnormal endothelium-derived NO level and impair cardiac function by disrupting the eNOS/NO pathway.

### 3.4. F-53B Alters eNOS and iNOS Expression and Phosphorylation

**Figure 4:**
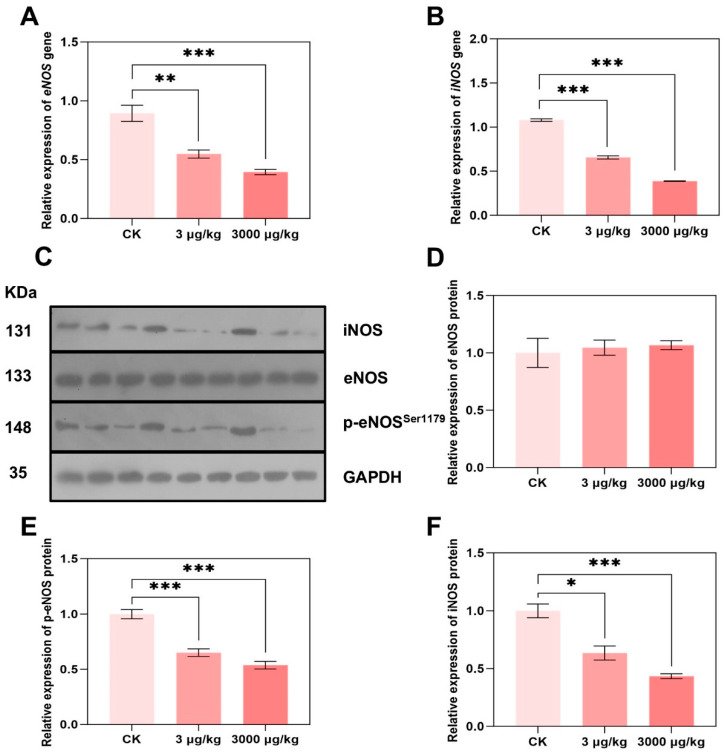
F-53B interfered expression and phosphorylation of iNOS, eNOS in parkin^−/−^ male mice. The relative mRNA level of *iNOS* and *eNOS* in the heart of parkin^−/−^ mice treated with 0, 3, 3000 μg/kg F-53B for 60 d were measured by qPCR (**A**,**B**), respectively (n = 3 biological replicates). The protein expression of iNOS, eNOS, and p-eNOS was measured and quantified by Western blot (**C**–**F**) (n = 3 biological replicates). All data are represented as means ± SEM. * represented *p* < 0.05, ** represented *p* < 0.01, and *** represented *p* < 0.001 compared with control group.

Despite the enzyme content, NO level was also affected by enzyme expression and phosphorylation of both eNOS and iNOS. To further elucidate the potential molecular mechanism of eNOS- and iNOS-derived NO, we examined the gene and protein expression of eNOS and iNOS, and phosphorylation of eNOS. At the transcriptional level, *eNOS* expression decreased to 0.61- and 0.36-fold compared with the control group (Figure 4A), and *iNOS* expression decreased to 0.61- and 0.44-fold after exposure of 3 and 3000 μg/kg, respectively (Figure 4B). Similarly, iNOS protein was significantly decreased to 0.63- and 0.43-fold of control group, respectively (Figure 4D). Total protein expression of eNOS showed no significant change in protein expression in both groups (Figure 4E), which is similar with our reported results of disturbed eNOS mRNA level but without effect on protein expression [31]. The phosphorylation of eNOS (p-eNOS^1179^), which serves as a representative regulatory mechanism for its activation [67], was further investigated under F-53B administration. However, both 3 and 3000 μg/kg of F-53B significantly reduced the stimulation of p-eNOS to 0.65- and 0.54-fold, respectively (Figure 4F), leading to the decrease in eNOS activity and NO production, which is in line with the results of decreased level of NO (Figure 3C).

Phosphorylation of eNOS increases eNOS enzyme activity and production of NO [68]. In this study, the content of eNOS and expression of p-eNOS was reduced in response to F-53B, implying that phosphorylation may be a key event in the down-regulation of eNOS content by F-53B. These results also showed that the content of iNOS enzyme increased, but gene and protein expression decreased. It is hypothesized that F-53B may inhibit the degradation pathway of iNOS protein [69], leading to the accumulation of the enzyme in the cell with elevated activity, but gene transcription is inhibited due to negative feedback or toxicity, and mRNA levels decrease, which needs to be further confirmed. Overall, these results suggest a disruptive potency of F-53B on cardiac endothelium function and NO level mainly via eNOS expression and phosphorylation at serine 1179.

## 4. Conclusions

In summary, this study revealed that environmentally relevant concentration of F-53B exposure for 60 d induced cardiac fibrosis and cardiac dysfunction in parkin^−/−^ mice. To the best of our knowledge, this is the first study that showed cardiac toxicity induced by co-effects of emerging PFAS, F-53B, and deficiency of cardiac function gene, parkin. Mechanistically, F-53B induced an abnormal level of NO, which is regulated by decreased content and phosphorylation of eNOS as well as increased level of iNOS. We emphasize that eNOS is a key molecule in F-53B which caused cardiotoxicity in parkin^−/−^ mice. In view of the cardiac regulation of parkin toward eNOS/NO signalling, these results further suggest that F-53B induced cardiac dysfunction via eNOS/NO pathway, indicating new mechanistic ideas for cardiac toxic chemicals. Further studies on cardiotoxicity induced by environmental chemicals including PFAS by comparison of parkin deficiency mice and wide type mice are warranted. In conclusion, our research results indicate that F-53B can cause structural and functional disorders of the heart, providing new toxicological data and more comprehensive health risk assessment of emerging PFAS.

Our study also has certain limitations. First, due to the limited availability of parkin^−/−^ mice, the number of biological replicates per assay was set at n = 3; increasing this sample size in future work would enhance the reproducibility of the data. Second, the current design lacks a wild-type (WT) mouse group. Incorporating a WT control group and directly comparing its responses with those of parkin^−/−^ mice would help to validate the gene–environment interaction (between the parkin gene and F-53B exposure) investigated here. Additionally, experimental methodologies could be further refined in subsequent studies; integrating functional assessments via cardiac echocardiography and quantifying F-53B concentrations in heart tissue would strengthen the rigour and comprehensiveness of the research.

## Data Availability

The data presented in this study are available on request from the corresponding author.

## Supplementary Materials

The following supporting information can be downloaded at: https://www.mdpi.com/article/10.3390/toxics13110942/s1, Figure S1: Cardiac morphology of male parkin^−/−^ mice after 7 d exposure to F-53B; Figure S2: Serum NO levels in male parkin^−/−^ mice exposed to F-53B (3 and 30 mg/kg BW/d, respectively) for 7 d (n = 3); Table S1: The primers used in qPCR.

## Abbreviations

The following abbreviations are used in this manuscript:

6:2 Cl-PFESA	6:2 Chlorinated Polyfluorinated Ether Sulfonate Acid
PFAS	Per- and Polyfluoroalkyl Substance
PFOS	Perfluorooctane Sulfonic Acid
PFOA	Perfluorooctanoic Acid
PFOSA	Perfluorooctane Sulfonamide
CVD	Cardiovascular Disease
eNOS	Endothelial Nitric Oxide Synthase
iNOS	Inducible Nitric Oxide Synthase
NO	Nitric Oxide
cTnT	Cardiac Troponin T
BNP	Brain Natriuretic Peptide
Mb	Myoglobin
ROS	Reactive Oxygen Species
p-eNOS	Phosphorylation of eNOS
WT	Wild-Type
