# Supplementary material for: Cardiotoxic Effect Induced by F-53B via Nitric Oxide Signalling on Parkin−/− Mice
Source: Toxics. 2025 Oct 31;13(11):942. doi: 10.3390/toxics13110942 (PMC12655989; doi:10.3390/toxics13110942)
Supplement: Supplementary file 1 [file toxics-13-00942-s001.zip › Supporting Information.pdf]

Supporting Information

Cardiotoxic effect induced by F-53B via nitric oxide signaling on Parkin$^{-/-}$ mice

Author: Jun Nie[a], Chao Hu[a], Yuru Huang[a], Ying Ma[a], Liping Lu [a], *.

[a] Hangzhou Normal University, Hangzhou, Zhejiang 311121, China.

* Address correspondence to E-mail: lipinglu@hznu.edu.cn

Table S1. The primers used in qPCR.

Gene	Primer Sequence (from 5' to 3')
GAPDH-F	GCCTCCTCCAATTCAACCCTT
GAPDH-R	TCACACCGACCTTCACCATT
iNOS-F	GACATTACGACCCCTCCCAC
iNOS-R	ACTCTGAGGGCTGACACAAG
eNOS-F	CTGGACATCACTTCCCCG
eNOS-R	GAGCTGGCTCATCCACGT

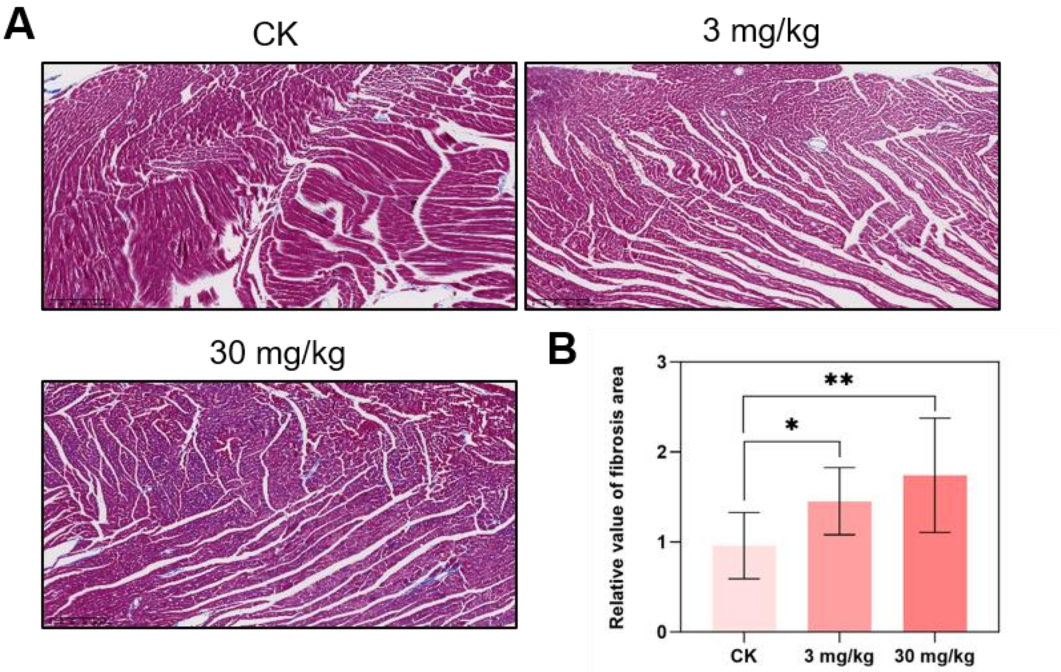

Figure S 1. Cardiac morphology of male parkin$^{-/-}$ mice after 7 d exposure to F-53B. Representative histological micrographs of the heart at control and F-53B groups (doses of 3 and 30 mg/kg BW/d, respectively). The scale bar is 200 μm. All data are represented as means ± SEM. * represented $p < 0.05$, ** represented $p < 0.01$ and *** represented $p < 0.001$ compared with control group.

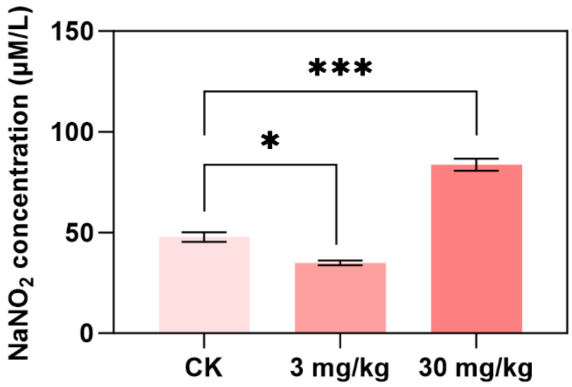

Figure S 2. Serum NO levels in male parkin$^{-/-}$ mice exposed to F-53B (3 and 30 mg/kg BW/d, respectively) for 7 d (n = 3). All data are represented as means ± SEM. * represented $p < 0.05$, ** represented $p < 0.01$ and *** represented $p < 0.001$ compared with control group.
